# Perceptions of the technical staff of professional teams regarding injury prevention in Spanish national futsal leagues: a cross-sectional study

**DOI:** 10.7717/peerj.8817

**Published:** 2020-03-25

**Authors:** Carlos Lago-Fuentes, Alejandro Jiménez-Loaisa, Alexis Padrón-Cabo, Marcos Mecías-Calvo, Ezequiel Rey

**Affiliations:** 1 Faculty of Education and Sport Sciences, Universidad de Vigo, Pontevedra, Spain; 2 Faculty of Health Sciences, Universidad Europea del Atlántico, Santander, Spain; 3 Department of Sport Sciences, Sport Research Center, Universidad Miguel Hernández de Elche, Elche, Spain

**Keywords:** Preventive strategies, Injury risk, Team sports, Tests, Sports injuries

## Abstract

Futsal is a sport with increasing popularity and level of performance, both in male and female categories. Also, there are several injuries along a season, so it is needed to know how to reduce this burden. The purpose of this study was to describe the perceptions of technical staff on injury risk factors, risk testing and preventive measures, and the strategies used by them within professional male and female futsal teams. A cross-sectional study was designed during the 2017–2018 season. A total of 32 futsal teams involved in male and female Spanish national futsal leagues completed, through an online survey platform, a questionnaire about injury risk factors, risk testing and preventive measures. Findings showed that: (a) most teams reported enough human resources, but insufficient material and time resources, (b) the main risk factors detected were previous injuries, strength deficits and dehydration, (c) functional movement patterns, flexibility tests and self-report questionnaires were the most applied tests for detecting injury risks in their players and (d) most of the main preventive measures used by technical staff matched with the best valued by them. Technical staff defined properly the main risk factors in futsal performance, as well as they applied preventive strategies with scientific support. The information provided in this research could be of interest for sport scientists and technical staff when designing more accurate and efficient injury prevention programs in futsal.

## Introduction

Sport injuries result in a negative impact on the individual and team performance, so they must be deeply studied for prevention (Van Mechelen, Hlobil & Kemper, 1992). Van Mechelen, Hlobil & Kemper (1992) presented the first model of sports injury prevention, which is based on a four-step process. Defining the injury incidence of a sport-specific population represent the first step, while understanding the etiology and mechanisms of the injury constitute the second step, respectively. Afterwards, the third step of the process is to include preventive measures focused on reducing the incidence and severity of injuries. Finally, the fourth step would assess the effectiveness of these preventive measures by repeating the first step (Van Mechelen, Hlobil & Kemper, 1992). Scientific evidence has shown that injury prevention programs (IPPs) have a positive effect on reducing the injury risk and injury incidence in many team sports (Monajati et al., 2016). Thus, determining factors related to specific injuries in each sport modality is needed to design efficient IPPs, which can imply a reduction in healthcare cost by teams (Marshall et al., 2016), as well as an improvement on sport performance (Faude et al., 2017; Thorborg et al., 2017; Owen et al., 2013). Most common IPPs are designed as multimodal exercise interventions in team sports, including contents about strength, balance or agility, among other measures (Emery et al., 2015). In this regard, the Fédération Internationale de Football Association (FIFA) 11+ has been shown to reduce overall injury risk by 39% in recreational and sub-elite football players (Thorborg et al., 2017). Other IPPs have shown a reduction in injury incidence in sports such as rugby, basketball or football (Owen et al., 2013; Al Attar et al., 2017; Brown et al., 2016; Pollard, Sigward & Powers, 2017).

In football, one of the most similar sports to futsal (Travassos, Araújo & Davids, 2018), McCall et al. (2014, 2015b) conducted various studies to know the differences between the evidence-based practices on injury prevention and the reality of elite football teams. In this sense, they studied the injury prevention strategies applied by staff members in European top football teams and replicated it with 32 national teams which participated at the 2014 FIFA World Cup, highlighting that injury risk perceptions, testing procedures, and preventive exercises were heterogeneous (McCall et al., 2015b). Following these studies, Meurer, Silva & Baroni (2017) designed a questionnaire to gain an in-depth knowledge about the specific preventive strategies used by physiotherapists and technical staff of Brazilian premier league football teams, finding that similar injury prevention strategies were applied within football clubs. Nevertheless, several studies found a gap between preventive practices used and the scientific evidence published (Meurer, Silva & Baroni, 2017; Wilke et al., 2018; Zech & Wellmann, 2017).

In futsal, three studies have analyzed the effects of FIFA 11+ on youth (Reis et al., 2013) and amateur teams (Lopes et al., 2019a, 2019b), showing contradictory results about improvements in physical performance, balance and proprioception (Reis et al., 2013; Lopes et al., 2019a, 2019b). However, a multistation exercise program applied twice a week seemed to improve the proprioceptive ability, but not the vertical jump performance (Pérez-Silvestre et al., 2019). Furthermore, these studies did not analyze the influence of the FIFA 11+ or other IPPs on futsal injury rates. Considering the high number of injuries in professional female and male futsal teams (Gayardo, Matana & Da, 2012; Serrano et al., 2013; Nemčić, Sporiš & Fiorentini, 2016; Martinez-Riaza et al., 2017), its impact on individual and team performance (Naser, Ali & Macadam, 2017; Beato, Coratella & Schena, 2016; Beato et al., 2017), and the neutral effects of the preventive programs designed on futsal (Reis et al., 2013; Lopes et al., 2019a, 2019b), it is necessary to increase the knowledge about the preventive strategies applied to improve the effectiveness of these interventions.

To the authors’ knowledge, no studies have explored the preventive strategies used by coaches in futsal teams. Therefore, the purpose of this study was to describe the perceptions of technical staff on injury risk factors, risk testing and preventive measures, and the strategies used by them within professional male and female futsal teams. Previous studies carried out with staff belonging to different teams have defined the same main injury risk factors for the same sport (McCall et al., 2014; Meurer, Silva & Baroni, 2017; Wilke et al., 2018). Considering the results of similar studies in other team sports about these points, we hypothesized that (1) most of the teams are satisfied with their resources for injury prevention, (2) there is homogeneity among the perceptions of the different technical staff about injury the risk factors and (3) there is a gap between the scientific knowledge on injury prevention and strategies applied by futsal staff.

## Methods

### Study design

A cross-sectional study was designed. Staff from participating futsal teams answered several questions from a structured questionnaire related with their perceptions about injury risk factors, risk testing and preventive measures applied on their teams. All teams provided written informed consent before fulfilling the questionnaire. The procedures used in this study were in accordance with the Declaration of Helsinki and approved by the Institutional Review Committee (Faculty of Health Sciences of the Universidad Europea del Atlántico) (CEI-01/2019). The quality of the study was assessed using the “Strengthening the reporting of observational studies in epidemiology” (STROBE) (White et al., 2015). This study fulfills all the criteria of the STROBE scale except the item 9 (Appendix A1).

### Participants

A total of 110 futsal teams belonging to the first and second male and female Spanish national leagues (season 2017–2018) were invited to participate in this study. A total of 59 teams confirmed the reception of the survey, and 32 of them completed it later (29.1% of the population). Of these 32 teams, 12 were male teams (seven of first and five of the second league, respectively), and 20 were female teams (eleven of the first and nine of the second league, respectively).

### Procedure

A questionnaire designed by Meurer, Silva & Baroni (2017) was translated to Spanish and adapted to futsal by a panel of experts, with a mixture of experts in questionnaires (qualitative methods) and futsal. Then, a pilot study of the survey was performed with five professional teams (Read et al., 2018). Based on this study, some modifications were applied, increasing the number of questions (including training load parameters). Finally, the official questionnaire was composed of 22 questions (17 closed and 5 open ended questions), divided into four sections: (1) pre-season and in-season information, (2) technical staff, their academic degrees and their participation in the prevention program, (3) perceptions regarding risk factors and (4) injury risk tests and preventive measures (see Appendix A2). Regarding preventive measures, we asked about the strategies applied in practice by the technical staff, as well as those that they perceived as the most important to reduce injury risk (regardless of whether they could apply them or not). Consequently, we could check if the preventive strategies used by teams matched with the best valued by them. The researchers contacted the 59 interested teams by phone, email or through national teams and players’ associations. The purpose of this contact was to explain the aims and procedures of the study to the contact person of each technical staff, as well as the privacy of data. However, 32 teams finally agreed to complete the survey. After accepting to participate, the survey was administered online to strength and conditioning coaches (main contact given by teams), being fulfilled with the consensus of all the members of staff (Survey Monkey, http://www.surveymonkey.net), as performed by similar studies (McCall et al., 2015b; Read et al., 2018). All data were collected between October 2017 and February 2018.

### Statistical analysis

Data were downloaded, codified and included in Statistical Package for Social Sciences (IBM SPSS) for Windows (20.0; Armonk, NY, USA) for further analysis. Descriptive data were calculated by means and standard deviations (M ± SD), as well as frequency counts and percentages (%) for qualitative variables. The normality of the data was checked with the Shapiro–Wilk test. All variables followed a normal distribution, except for the variable “number of matches”. To calculate the relevance of each risk factor, the answers “very important”, “important” and “not important” were considered as two, one and zero points, respectively. Then, points were summed and ranked from the highest to the lowest score to indicate their importance. In addition, most effective exercises used in the IPPs were graded using a 5-point Likert scale, in accordance with earlier studies (McCall et al., 2014; Meurer, Silva & Baroni, 2017), where “5” indicated the exercise perceived as the most important, and “1” the least important.

## Results

### Pre-season and in-season data

The data of pre- and in-season periods are shown in Table 1. The pre-season lasted 30 days on average for all teams. Most teams played around 40 matches per season.

**Table 1:** Descriptive data of external load during pre-season and in-season of professional Spanish futsal teams.

	Total sample
	Mean ± SD (95% confidence interval)
Training days pre-season	30.1 ± 7.8 [28.0–33.6]
Training sessions pre-season	28.3 ± 12.8 [23.7–32.9]
Training days per week season	3.8 ± 1.1 [3.5–4.2]
Training sessions per week season	4.4 ± 1.9 [3.7–5.1]
Matches pre-season	5.0 ± 1.7 [4.4–5.6]
Matches during the season	38.5 ± 3.6 [37.2–39.7]

### Staff members, qualifications and roles

Staff were composed of 2–6 employees, most of whom participated actively during the IPPs (Table 2). Most teams (87.5%) had, at least, a head coach, a second coach, a strength and conditioning coach (S&C) and a physiotherapist. Generally, the 71.9% of the teams were satisfied with their human resources for injury prevention, but only the 37.5% and 40.6% were satisfied with their material and temporal resources on this matter, respectively.

**Table 2:** Staff involved in the injury prevention programs of professional Spanish futsal teams.

	Medical doctor	Strength and conditioning coach	Physiotherapist	Injury specialist	Head coach	Second coach	Assistant	Psychologist
Highest academic degree (all teams)*								
No high education	–	1 (3.6%)	–	2 (16.7%)	4 (12.5%)	4 (14.3%)	8 (40.0%)	2 (22.2%)
VE	–	3 10.7%)	1 (3.6%)	1 (8.3%)	11 (34.4%)	10 (35.7%)	6 (30.0%)	–
Undergrad	5 (31.3%)	8 (28.6%)	17 (60.7%)	4 (33.3%)	11 (34.4%)	12 (42.9%)	6 (30.0%)	2 (22.2%)
MSc	2 (12.5%)	16 (57.1%)	10 (35.7%)	5 (41.7%)	6 (18.8%)	2 (7.1%)	–	4 (44.4%)
PhD	9 (56.3%)	–	–	–	–	–	–	1 (11.1%)
Total	16	28	28	12	32	28	20	9
Roles in the prevention program								
Design	1 (9.1%)	2 (7.1%)	2 (7.7%)	2 (16.7%)	2 (10.0%)	1 (6.7%)	1 (25.0%)	3 (50.0%)
Testing	8 (72.7%)	–	2 (7.7%)	–	2 (10.0%)	3 (20.0%)	–	1 (16.7%)
Application	–	2 (7.1%)	4 (15.4%)	1 (8.3%)	5 (25.0%)	5 (33.3%)	3 (75.0%)	1 (16.7%)
Involved in 2 stages	2 (18.2%)	4 (14.2%)	5 (19.2%)	1 (8.3%)	2 (10.0%)	1 (6.7%)	–	1 (16.7%)
Involved in 3 stages	–	20 (71.4%)	13 (50.0%)	8 (66.7%)	9 (45.0%)	5 (33.3%)	–	–

**Note:**

* VE, vocational education; MSc, master’s degree; PhD, doctorate.

### Perceived injury risk factors and screening tests

**Figure 1:**
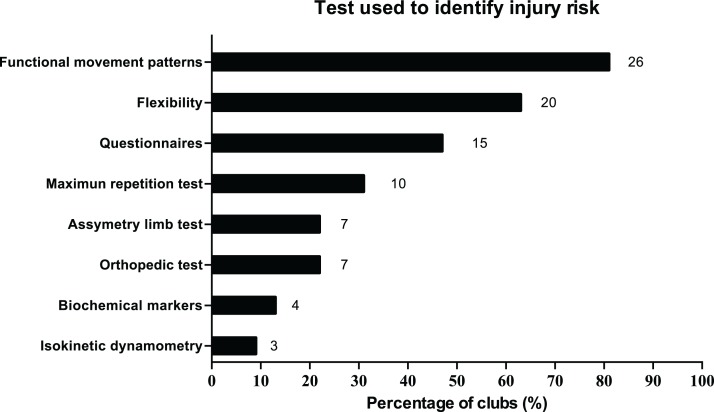
Tests used to identify injury risk.

The main risk factors perceived by technical staff to suffer an injury were having a previous injury, strength deficits and dehydration (Table 3). Having a previous injury and strength deficits were valued equally by participants. Regarding screening tests, functional movement patterns, flexibility tests and self-report questionnaires were the most used during the pre-season periods (Fig. 1).

**Table 3:** Perception of team staff regarding the importance of specific risk factors for non-contact injuries in futsal.

Risk factors	Not important	Important	Very important	Accumulated score (maximum points = 64)
Previous injury	–	13 (40.6%)	19 (59.4%)	51
Strength deficit	–	13 (40.6%)	19 (59.4%)	51
Hydration	1 (3.1%)	13 (40.6%)	18 (56.3%)	49
Muscle imbalance	1 (3.1%)	14 (43.8%)	17 (53.1%)	48
Diet	–	17 (53.1%)	15 (46.9%)	47
Sleep/rest	1 (3.1%)	15 (46.9%)	16 (50.0%)	47
Conditioning	–	17 (53.1%)	15 (46.9%)	47
Fatigue	1 (3.1%)	17 (53.1%)	14 (43.8%)	45
Kind of surface	2 (6.3%)	17 (53.1%)	13 (40.6%)	43
Anatomy/morphology	2 (6.3%)	22 (68.8%)	8 (25.0%)	38
Flexibility	7 (21.9%)	15 (46.9%)	10 (31.1%)	35
Genetics	4 (12.5%)	22 (68.8%)	6 (18.8%)	34
Psychological factors	4 (12.5%)	24 (75.0%)	4 (12.5%)	32
Futsal boots	8 (25.0%)	16 (50.0%)	8 (25.0%)	32
Temperature	6 (18.8%)	23 (71.9%)	3 (9.4%)	29
Blood markers	12 (37.5%)	18 (56.3%)	2 (6.3%)	22

### Frequency of the IPPs

A total of 31 staff stated that IPPs could reduce the number of injuries (97% of teams), and also applied IPPs. A total of 23 (74.1%) of the technical staff applied both individual and global IPPs, where 6 (19.4%) and 2 (6.5%) used only global and individual preventive programs, respectively. One team reported that they have not designed or implemented an IPP. However, this team included some preventive exercise once per week during the in-season period. The frequency by which IPPs were applied by technical staff is shown in Table 4.

**Table 4:** Weekly frequency of preventive programs during the season of professional Spanish futsal teams.

	No IPP	<1×/week	1×/week	2×/week	3×/week	>3×/week
Pre-season	1 (3.1%)	3 (9.4%)	7 (21.9%)	10 (31.3%)	4 (12.5%)	7 (21.9%)
Season: 1 Match/week	–	7 (19.4%)	11 (35.5%)	9 (28.1%)	3 (9.7%)	2 (6.4%)
Season: 2 Matches/week	18 (56.3%)	2 (6.3%)	8 (25.0%)	2 (6.3%)	2 (6.3%)	–

**Note:**

– No teams.

### Perceived and applied injury preventive measures

**Figure 2:**
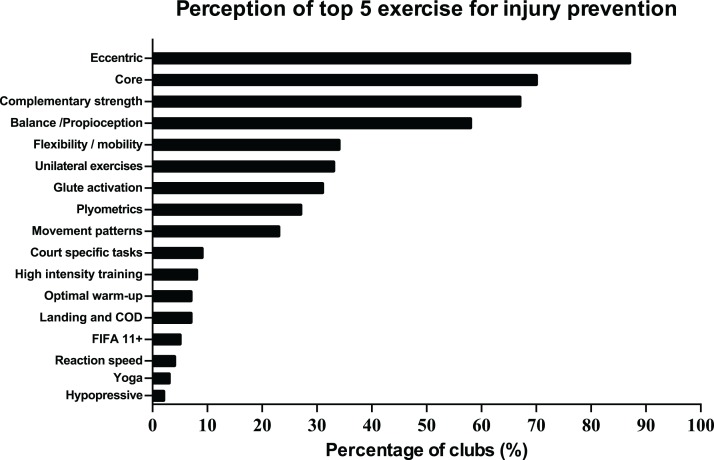
Perceptions of technical staff about the top 5 exercises for injury prevention.

**Figure 3:**
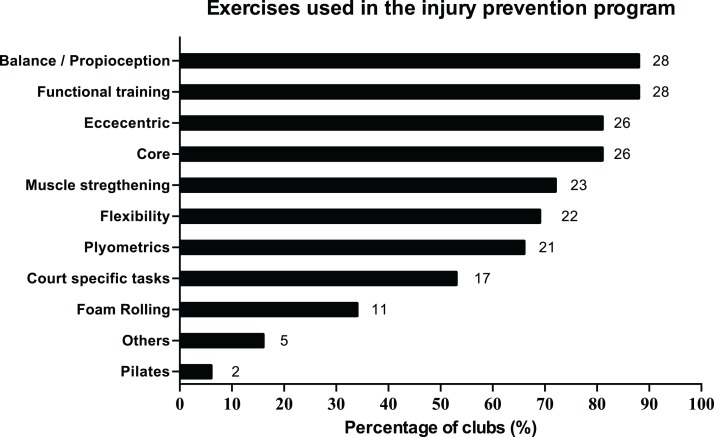
Frequency of the most common exercises used in the injury prevention programs.

The most common preventive measures applied in IPPs by staff were balance/proprioception exercises, functional training, eccentric and core exercises (Fig. 2). Eccentric and core exercises obtained the same score (81.3%). When asked about perceived preventive measures, technical staff ranked eccentric, core exercises and complementary strength were the most important for them (Fig. 3). Accordingly, 81.3% of teams included these exercises in their preventive programs. Balance/proprioception exercises and functional training, which were the most used prevention measures, also were perceived as relevant strategies to reduce injury risk by technical staff (90%) (Fig. 3). Regarding training load management, staff indicated that intensity, variety of exercises and volume of training were useful variables to injury prevention (94.0%, 91.0% and 84.0% respectively).

## Discussion

To date, this is the first study which explores the perceptions of the staff members of professional futsal teams about preventive strategies and their application. The goal of the study was to describe the perceptions and strategies regarding injury risk factors, risk testing and preventive measures within professional male and female futsal teams. Generally, findings showed that: (a) most teams reported enough human resources, but insufficient material and time resources, (b) main risk factors detected were previous injuries, strength deficits and dehydration, (c) movement patterns, flexibility tests and self-report questionnaires were the most applied tests for detecting injury risks in their players and (d) most of the main preventive strategies used by technical staff matched with the best valued by them.

### Staff members, qualifications and roles

A total of 23 teams (71.9%) reported enough human resources for injury prevention, which in turn participated actively in different phases of the IPPs. However, it should be noted that only 10 teams had PhDs in their technical staff, and none of them were strength and conditioning coaches, physiotherapists, or injury specialists. Including the perceptions and practices of highly qualified personnel within the sports teams could be important to develop IPPs based on scientific evidence. Meurer, Silva & Baroni (2017) found that most views of professional football practitioners regarding risk factors, screening tests and preventive exercises were not supported by inquiry. In this regard, further research is required to explore the relationships between staff qualifications, management of injury risk factors, and injury incidence and burden. On the other hand, more than the 50% of the teams reported insufficient material and temporal resources. More support from sports federations, leagues, sponsors and public institutions is needed to provide clubs with resources that directly or indirectly care for the health of athletes. Clubs should also be aware of the importance of having qualified staff among their teams.

### Perceived injury risk factors

Staff highlighted previous injuries, strength deficits and dehydration as the main risk factors for non-contact injuries, similarly to results from other studies with football technical staff (Meurer, Silva & Baroni, 2017; McCall et al., 2015a), and about perceptions of football players (Zech & Wellmann, 2017). Previous injury is usually considered as an important injury risk factor confirmed by a high level of evidence (McCall et al., 2015a). In fact, a recent study (Ruiz-Pérez et al., 2019) on female futsal players showed that 20% of recurrent injuries occurred within 2 months after return to play. For that reason, this factor should be controlled by technical staff by designing individualized preventive strategies for each player (Jiménez-Rubio et al., 2019) that ensure adequate rehabilitation and return to train/play processes. Strength deficits are also reported in previous studies (Meurer, Silva & Baroni, 2017; Wilke et al., 2018), which highlights the importance to design training plans based on neuromuscular inputs with, at least, more than 20 sessions during the in-season period to improve muscular strength and to reduce muscular imbalances (Faude et al., 2017). In this sense, some studies have shown the importance of managing muscular imbalances to reduce the injury risk and to improve the return to train/play times, especially in the case of hamstring injuries (Jiménez-Rubio et al., 2019; Mendiguchia et al., 2017; Navarro et al., 2015). Additionally, previous studies reported dehydration as a critical factor in futsal performance (García Jiménez et al., 2011, 2015). Considering that even modest dehydration have negative effects on aerobic performance (Barr, 1999), having available water or sport drinks during the training and matches sessions could be essential to reduce the risk of dehydration and, consequently, the injury risk (García Jiménez et al., 2011, 2015). Fatigue has been reported as one of the main risk factors in other studies (McCall et al., 2015b; Meurer, Silva & Baroni, 2017), but with some contradictory results (McCall et al., 2015a). In our study, this factor was not perceived as especially important (eighth in the ranking), a fact that could be related to the unlimited substitutions allowed during the futsal matches, which allows teams to maintain rhythm and intensity throughout the match while players are substituted to rest (Naser, Ali & Macadam, 2017).

### Screening tests

The most common tests to identify possible injury risks were those related with functional movement patterns, flexibility and self-report questionnaires. Our results are far from the results by Meurer, Silva & Baroni (2017) in professional Brazilian football clubs, where biochemical markers and isokinetic dynamometry were the two main tests used, while movement patterns and flexibility tests were fourth and fifth, respectively. Meanwhile, our results are similar to the study of McCall et al. (2015b) with 44 professional football teams, showing functional movement patterns tests (e.g., the Functional Movement Screen (FMS)) and self-report questionnaires as the ones most frequently used. Regarding movement patterns tests, staff should be aware of their limitations to predict injury risks according to the final score, as suggested for the FMS (Moran et al., 2017). Curiously, biochemical tests were poorly applied by the sample of our study, similar to previous studies with European teams (McCall et al., 2014; Zech & Wellmann, 2017). Respecting to flexibility tests, these have been widely used in basketball and football national teams (McCall et al., 2015b; Wilke et al., 2018). However, there is no conclusive evidence to support stretching to prevent injuries (McCall et al., 2015b; Lewis, 2014). For their part, teams of this study indicated some self-report questionnaires such as Profile of Mood States, the Recovery-Stress Questionnaire for Athletes and the perceived muscle soreness scale to detect injury risk in their players. Although these types of instruments could be important indicators of the athletes’ recovery states, there is insufficient evidence to ensure their validity as markers of injury risk (Meurer, Silva & Baroni, 2017). Further studies should explore what psychological factors constitute risk factors, in order to elaborate future questionnaires that help to identify players who show these specific psychological risk factors (Meurer, Silva & Baroni, 2017).

### Perceived and applied preventive measures

Staff applied some of the same measures they perceived as the most efficient to reduce injury risk. However, the order of importance as its frequency of use in the IPPs was different. Most of the main preventive strategies used match with previous studies in football (McCall et al., 2014, 2015b; Meurer, Silva & Baroni, 2017; Wilke et al., 2018). Interestingly, scientific evidence has attested an appropriate combination of different exercises as the best strategy to prevent injuries with team sport athletes (Monajati et al., 2016; Faude et al., 2017), showing improvements on neuromuscular ability and reducing the injury risk after applying IPPs. These programs should include a special emphasis on neuromuscular training, including balance, agility and strength, among other preventive strategies (Emery et al., 2015).

Eccentric exercises were perceived as the most effective preventive strategy and as the third most commonly used, similarly to other studies (McCall et al., 2014, 2015b; Meurer, Silva & Baroni, 2017). This strategy has been well supported by scientific evidence given its role preventing injuries. Eccentric exercises can be especially interesting for male futsal teams due to the higher incidence of hamstring injuries (Martinez-Riaza et al., 2017), when compared with female teams (Gayardo, Matana & Da, 2012; Serrano et al., 2013; Nemčić, Sporiš & Fiorentini, 2016). The second perceived preventive strategy was core training, according to previous research carried out in football (McCall et al., 2014; Meurer, Silva & Baroni, 2017). Poor trunk stability has been signaled as a risk factor in lower limb injuries (Meurer, Silva & Baroni, 2017). Core exercises have the goal to improve trunk stability and optimize coordination between upper and lower limbs in fast actions (Meurer, Silva & Baroni, 2017). Despite this argument, more studies are needed to evaluate the association of well trunk stabilization and low risk of injury. Complementary strength was also perceived as an important preventive measure to reduce injury risk. Strength work is a fundamental axis when it comes to preventing sport injuries and must be included in IPPs by technical staff. In a meta-analysis, Lauersen, Bertelsen & Andersen (2014) showed how strength training reduced injuries to less than 1/3, decreasing overuse injuries by half. In futsal, experimental studies which explore the effectiveness of strength-based interventions on this matter are needed. Finally, balance/proprioception was one of the most common strategies valued and applied in IPPs by our teams. These exercises seem to be effective to prevent ankle sprains (McCall et al., 2015a). However, there is not a consensus about its influence in other type of injuries (Wilke et al., 2018; McCall et al., 2015a). Some other strategies, such as hypopressives, were also ranked as efficient in reducing injury risk. In this line, other studies highlighted the use of several preventive strategies, such as hypopressives among others, lacking scientific evidence on their effectiveness on injury prevention (Wilke et al., 2018; Zech & Wellmann, 2017; Martín-Rodríguez & Bø, 2017). This can also be explained by low quality of some studies about injury prevention which makes harder to know which strategies are better to use in each context (McCall et al., 2015a). For all these reasons, linked to the low number of PhD in technical staff and the use of some preventive strategies without scientific evidence, more scientific profiles are needed in staff to transfer the scientific evidence to the court. That is, there is a need for designing individualized programs on how to introduce evidence-based injury prevention strategies to the technical staff and, in turn, to the players (Wilke et al., 2018). Moreover, these strategies have to be adapted to specific characteristics of the sport, sex, age and performance level to ensure the specificity of the injury prevention strategies (Faude et al., 2017; Meurer, Silva & Baroni, 2017; Wilke et al., 2018; Zech & Wellmann, 2017; McCall et al., 2015a).

Finally, most of the teams only apply IPPs once or twice a week during the pre-season, and once or less during the competitive season, which is less frequent than in other studies (Read et al., 2018). However, frequency of IPPs during the week will depend of characteristics of team, density of competition or travels, among other factors. So, further studies should compare the efficiency of IPPs according to the different weekly frequencies.

### Strengths and limitations

To the authors’ knowledge, this is the first study which has explored the perceptions of the staff members about preventive strategies and their application in futsal. Future research could use these results to explore the explained variance of each risk factor for injury in futsal, as well as to explore what are the most effective interventions to reduce these sport-specific factors. In this regard, future prospective studies are needed to discuss how perceived risk factors are statistically related to the measured risk factors in futsal. On the other hand, our study has also some limitations which have to be considered. First, we were limited by the cross-sectional design chosen to conduct this study, which impaired us to establish causal relationships. Second, the collected data provided us a snapshot of the injury risk factors, risk testing and preventive measures at a single point in time, so we cannot ensure that our results are free of bias when filling out the questionnaire by the teams. Third, the questionnaire only asked about injury prevention strategies, but no about whether multimodal IPPs were applied and the elements that conformed them. Finally, information about the injuries during the pre-season period was included, but we did not get deep information about them to be able to analyze possible relations between preventive measures and suffered injuries.

## Conclusions

The findings of the present study suggest that staff members participated actively in different phases of the IPPs. Staff perceived a previous injury, presenting muscular strength deficits and dehydration as the main injury risk factors in futsal. Interestingly, functional movement patterns, flexibility tests and self-report questionnaires were the most common tools to evaluate possible injury risks during the season. Teams were coherent with the perceptions and strategies of injury prevention, applying the same exercises in their programs as they thought these were the most effective. The information provided in this paper could be of interest for sport scientists and futsal technical staff when designing more accurate and efficient IPPs.

## Supplemental Information

10.7717/peerj.8817/supp-1 Supplemental Information 1 STROBE checklist.

10.7717/peerj.8817/supp-2 Supplemental Information 2 Questionnaire used on the research.

10.7717/peerj.8817/supp-3 Supplemental Information 3 Results obtained on the questionnaire.

## Additional Information and Declarations

### Data Availability

The following information was supplied regarding data availability: The questionnaire is available as a Supplemental File.
