# Supplementary material for: Perceptions of the technical staff of professional teams regarding injury prevention in Spanish national futsal leagues: a cross-sectional study
Source: PeerJ. 2020 Mar 25;8:e8817. doi: 10.7717/peerj.8817 (PMC7102501; doi:10.7717/peerj.8817)
Supplement: Supplemental Information 2 [file peerj-08-8817-s002.docx]

**STRATEGIES FOR INJURY PREVENTION IN SPANISH FUTSAL: PERCEPTION OF THE TECHNICAL STAFFS** o**F MALE AND FEMALE FUTSAL TEAMS**

| Club name: | |
| --- | --- |
| City: | Province: |
| Category: | |
| Responsible: | |
| Telephone: | |
| E-mail: | |
| Date of answer: | |

**Part 1: Pre**–**season/season information:**

1. How many pre–season days did your team have this year?

| DAYS |  |
| --- | --- |

1. How many training sessions did your team have?

| TRAINING SESSIONS |  |
| --- | --- |

1. How many days does your team train per week during the in–season period?

| DAYS |  |
| --- | --- |

1. How many training sessions did the team have during the week?

| TRAINING SESSIONS |  |
| --- | --- |

1. Complete the table below with the number of played matches in each competition throughout the last pre–season.

| COMPETITION | MATCHES |
| --- | --- |
|  |  |
|  |  |
|  |  |
|  |  |
|  |  |
|  |  |

Observations:

|  |
| --- |

1. Did the team suffer any injuries during the pre–season?

| YES |  |
| --- | --- |
| NO |  |

1. If so, how many injuries did the team have?

| NUMBER OF INJURIES |  |
| --- | --- |

1. By mean, how many days were the players injured?

| MEAN OF DAYS |  |
| --- | --- |

1. Are you satisfied with the strategies used to preventing injuries in your team?

| YES |  |
| --- | --- |
| NO |  |

1. Do you believe your team have sufficient resources to measure and quantify precisely the injury risk factors of your players?

|  | HUMAN RESOURCES | MATERIAL RESOURCES | TIME  RESOURCES |
| --- | --- | --- | --- |
| YES |  |  |  |
| NO |  |  |  |

**Part 2: Technical staff involved in the injury prevention program.**

1. Please specify each professional of your staff involved in the injury prevention program (e.g., physiotherapist, medical doctor, conditioning coach, coach, second coach, etc.), his/her highest academic degree (e.g., undergrad, specialist, masters, PhD), and his/her involvement with each phase of the prevention program (design, testing and application).

| PROFESSIONAL | DEGREE | DESIGN | TESTING | APPLICATION |
| --- | --- | --- | --- | --- |
|  |  |  |  |  |
|  |  |  |  |  |
|  |  |  |  |  |
|  |  |  |  |  |
|  |  |  |  |  |
|  |  |  |  |  |
|  |  |  |  |  |
|  |  |  |  |  |

Observations:

|  |
| --- |

**Part 3: Perceptions of technical staff on risk factors for non-contact injuries.**

1. Mark the risk factors below as “very important”, “important” or “not important”.

| RISK FACTOR | VERY  IMPORTANT | IMPORTANT | NOT  IMPORTANT |
| --- | --- | --- | --- |
| Previous injury |  |  |  |
| Fatigue |  |  |  |
| Strength deficits |  |  |  |
| Anatomy/Morphology |  |  |  |
| Genetics |  |  |  |
| Muscular imbalances |  |  |  |
| Sleep / Rest |  |  |  |
| Flexibility |  |  |  |
| Psychological factors |  |  |  |
| Conditioning |  |  |  |
| Blood markers |  |  |  |
| Futsal boot |  |  |  |
| Dehydration |  |  |  |
| Diet |  |  |  |
| Kind of surface |  |  |  |
| Temperature |  |  |  |

1. List any risk factor perceived in your practice that are not included in the previous table, and mark it as “very important”, “important” and “not important”.

| RISK FACTOR | VERY  IMPORTANT | IMPORTANT | NOT  IMPORTANT |
| --- | --- | --- | --- |
|  |  |  |  |
|  |  |  |  |
|  |  |  |  |
|  |  |  |  |
|  |  |  |  |
|  |  |  |  |
|  |  |  |  |

Observations:

|  |
| --- |

**Part 4. Tests used to identify players’ injury risk.**

1. When were the injury risk screening tests performed on your team in the last year?

| Only during pre–season |  |
| --- | --- |
| Only during in–season |  |
| During both pre–season and in–season |  |

1. Mark screening tests to value the injury risk used this year:

| Functional pattern tests |  | Others: |  |
| --- | --- | --- | --- |
| Isokinetic dynamometry |  | Others: |  |
| Orthopedic tests |  | Others: |  |
| Flexibility |  | Others: |  |
| Biochemical markers |  | Others: |  |
| Questionnaires |  | Others: |  |
| Maximal repetitions |  | Others: |  |
| Asymmetry tests |  | Others: |  |

Observations:

|  |
| --- |

1. Do you believe that an injury prevention program is able to reduce the injury rate in professional futsal players?

| YES |  |
| --- | --- |
| NO |  |

1. Do players perform an injury prevention program on your team?

| YES |  |
| --- | --- |
| NO |  |

If NO, explain the main reason(s). Then, go to question 21

1. How the injury prevention program is prescribed for players?

| Global (all players follow the same program) |  |
| --- | --- |
| Individual (players follow specific programs) |  |
| Both global and individual |  |

1. How often the injury prevention program is applied in the following situations?

During preseason:

| <1 x/ week |  | 4 x/ week |  |
| --- | --- | --- | --- |
| 1 x/ week |  | 5 x/ week |  |
| 2 x/ week |  | >5 x/ week |  |
| 3 x/ week |  |  |  |

During season, when team plays 1 match per week:

| <1 x/ week |  | 4 x/ week |  |
| --- | --- | --- | --- |
| 1 x/ week |  | 5 x/ week |  |
| 2 x/ week |  | >5 x/ week |  |
| 3 x/ week |  |  |  |

During season, when team plays 2 matches per week:

| <1 x/ week |  | 4 x/ week |  |
| --- | --- | --- | --- |
| 1 x/ week |  | 5 x/ week |  |
| 2 x/ week |  | >5 x/ week |  |
| 3 x/ week |  |  |  |

1. Mark the exercises currently used in the injury prevention program of your team:

| Gym exercises |  | Others: |  |
| --- | --- | --- | --- |
| Functional exercises |  | Others: |  |
| Plyometric |  | Others: |  |
| Pilates |  | Others: |  |
| Core (trunk stabilization) |  | Others: |  |
| Eccentric |  | Others: |  |
| Flexibility /mobility |  | Others: |  |
| Balance /proprioception |  | Others: |  |
| Foam rolling |  | Others: |  |
| Court specific tasks |  | Others: |  |

1. Based on your perception, rank in order of importance the 5 most effective exercises for injury prevention in futsal players:

| CLASSIFICATION | EXERCISES |
| --- | --- |
| 1^ST^ |  |
| 2^ND^ |  |
| 3^RD^ |  |
| 4^TH^ |  |
| 5^TH^ |  |

1. Mark the aspects taken into account to design the injury prevention program of your team:

| Execution velocity |  | Others: |  |
| --- | --- | --- | --- |
| Variety of exercises |  | Others: |  |
| Intensity |  | Others: |  |
| Volume |  | Others: |  |
| Density |  | Others: |  |
